# Accelerometric Trunk Sensors to Detect Changes of Body Positions in Immobile Patients

**DOI:** 10.3390/s18103272

**Published:** 2018-09-28

**Authors:** Katrin Rauen, Judith Schaffrath, Cauchy Pradhan, Roman Schniepp, Klaus Jahn

**Affiliations:** 1 Schoen Clinic Bad Aibling, Kolbermoorer Strasse 72, 83043 Bad Aibling, Germany; judym.s.83@gmail.com (J.S.); klaus.jahn@med.uni-muenchen.de or kljahn@schoen-kliniken.de (K.J.); 2 Institute for Regenerative Medicine, University of Zurich, Wagistrasse 12, 8952 Schlieren, Switzerland; 3 Institute for Stroke and Dementia Research, University of Munich Medical Center, Feodor-Lynen-Strasse 17, 81377 Munich, Germany; 4 German Center for Vertigo and Balance Disorders, University of Munich Medical Center, Marchioninistrasse 15, 81377 Munich, Germany; cauchypradhan@gmail.com (C.P.); roman.schniepp@med.uni-muenchen.de (R.S.); 5 Department of Neurology, University of Munich Medical Center, Marchioninistrasse 15, 81377 Munich, Germany

**Keywords:** accelerometric trunk sensors, accelerometry, changes of body positions in immobile patients, early neurorehabilitation, monitoring mobilization frequency, polyneuropathy, quantification of body position and mobility, traumatic brain injury, stroke

## Abstract

Mobilization, verticalization and position change are mandatory for severely affected neurological patients in early neurorehabilitation in order to improve neurological status and prevent complications. However, with the exception of hospitals and rehabilitation facilities, this activity is not usually monitored and so far the automated monitoring of position changes in immobile patients has not been investigated. Therefore, we investigated whether accelerometers on the upper trunk could reliably detect body position changes in immobile patients. Thirty immobile patients in early neurorehabilitation (Barthel Index ≤ 30) were enrolled. Two tri-axial accelerometers were placed on the upper trunk and on the thigh. Information on the position and position changes of the subject were derived from accelerometer data and compared to standard written documentation in the hospital over 24 h. Frequency and duration of different body positions (supine, sidelying, sitting) were measured. Data are presented as mean ± SEM. Groups were compared using one-way ANOVA or Kruskal-Wallis-test. Differences were considered significant if *p* < 0.05. Trunk sensors detected 100% and thigh sensors 66% of position changes (*p* = 0.0004) compared to standard care documentation. Furthermore, trunk recording also detected additional spontaneous body position changes that were not documented in standard care (81.8 ± 4.4% of all position changes were documented in standard care documentation) (*p* < 0.0001). We found that accelerometric trunk sensors are suitable for recording position changes and mobilization of severely affected patients. Our findings suggest that using accelerometers for care documentation is useful for monitoring position changes and mobilization frequencies in and outside of hospital for severely affected neurological patients. Accelerometric sensors may be valuable in monitoring continuation of care plans after intensive neurorehabilitation.

## 1. Introduction

Early neurorehabilitation in severe neurological disorders, e.g., following stroke, traumatic brain injury or critical illness polyneuropathy, is characterized by loss of functional independence and is usually measured with a Barthel Index ≤ 30, representing major activity limitations in the patient’s daily life. These patients suffer from severe motor deficits and are not able to move independently. Furthermore, most patients show reduced alertness and cognitive deficits which limit their capability to notice the need for positioning change themselves or to ask nursing staff or caregivers for repositioning. Consequently, patients are dependent on positioning on a regular basis from their caregivers. It is well-known that regular positioning and passive physical activity improve function and general health, decrease complications, contribute to regaining patients’ independence, and reduce mortality [1,2,3,4,5]. Thus, mobilization and positioning are important contributors to a good functional outcome for severely affected immobile patients.

In hospitals and rehabilitation facilities, changes of body position are regularly undertaken by the nursing staff as part of the treatment and care plan during early neurorehabilitation. Frequency of position change and duration of mobilization is documented manually in the patient’s charts. However, mobilization outside the hospital, in nursing homes or at home, is not usually monitored. Approximately one third of the patients in neurorehabilitation experience re-hospitalization due to infection, neurological deterioration, or other reasons and acute readmission within 30 days after discharge is a major issue [6,7,8,9]. To date it remains unclear if improved positioning and mobilization post discharge might reduce re-hospitalization rates. Therefore, we followed the hypothesis that frequency and duration of mobilization correlate with better functional outcome, and that thus automated assessment of motor activity, positioning, and mobilization would be helpful. Interventions to ensure optimal mobilization would thus help to improve patients’ long-term outcomes.

**Figure 1:**
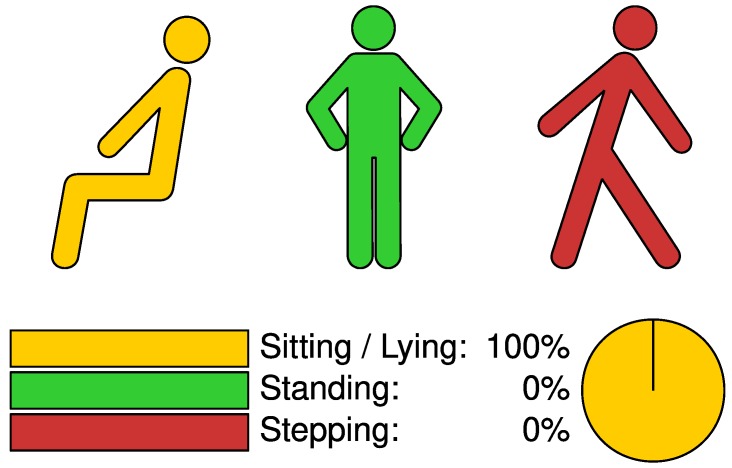
**Automated accelerometric data recording of an immobile patient during early neurorehabilitation**. An 81-year-old man suffering from polytrauma with severe traumatic brain injury six weeks prior to automated accelerometric recording over 24 h. Neurological status: Tetraparesis, tracheostomy due to severe dysphagia, cognitive deficits including lack of alertness and complete incontinence with a Barthel Index of 10 indicating complete dependency on staff and caregivers. No ability to independently turn or move. Automatic accelerometric recording does not distinguish position changes of this immobile patient during early neurorehabilitation.

Accelerometers have been increasingly used to assess physical activity outside of hospitals and in gait laboratories mainly by distinguishing between walking and sedentary behavior, e.g., in stroke patients and older adults [10,11,12,13]. Current sensors can store data for long periods of time (weeks), are user-friendly and detect valid and reliable body accelerations in walking populations. They are affordable and robust enough to be used in many different environments and are thereby most suitable for detecting mobilization characteristics in neurological patients during and after discharge from hospital care [10,12,13,14,15]. Multi-sensor approaches have been utilized to characterize the link between trunk accelerations and walking, and this approach have detected, e.g., the lack of pelvic accelerations and impaired head position or upper extremity function. Moreover, accelerometric analysis of trunk movements has been suitable for distinguishing stroke patients from healthy controls due to their trunk asymmetry and gait disturbance, with good correlations with clinical balance and mobility scores [11,13,15,16,17,18,19,20]. However, to date, there is no automated monitoring of position changes in early neurorehabilitation available, and accelerometric sensors have not been used to assess changes of body position in immobile patients during early neurorehabilitation. Until now, the automatic accelerometric data recording of immobile patients cannot distinguish position changes (Figure 1). Furthermore, thigh position changes less often than trunk position due to the regular positioning in supine, side-lying or sitting of immobile patients due to their loss of function. Thus, the aim of the current study was to investigate whether accelerometers—positioned on the upper trunk—can reliably detect changes in body position in immobile patients during early neurorehabilitation.

## 2. Materials and Methods

### 2.1. Accelerometers and Computation

**Figure 2:**
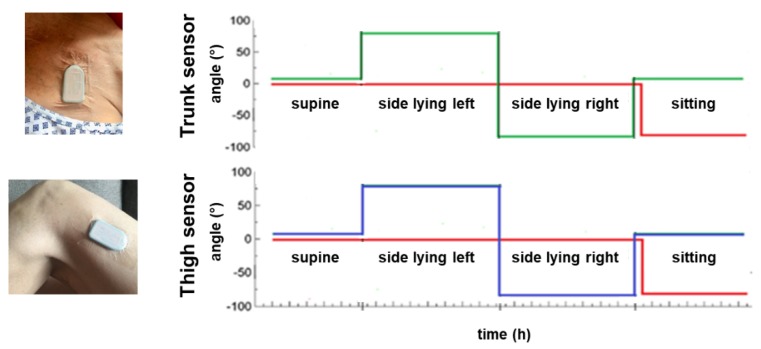
**Schematic accelerometric recording of trunk and thigh sensors**. The given angles define and distinguish the positions of supine, side-lying left/right or sitting in immobile patients during early neurorehabilitation (*green*: transversal axis (x), *red*: longitudinal axis (y), *blue*: sagittal axis (z)). To distinguish these four positions in immobile patients, two axes are necessary per sensor due to the sensor’s perpendicular position in the frontal plane on the upper trunk and in the sagittal plane on the lateral thigh. Thus, the transversal and the sagittal axes are interchangeable between the trunk and the thigh sensor.

Two activPAL micro™ tri-axial accelerometers (PAL Technologies, Scotland, UK) were used to record the patient’s body orientation and movement by measuring accelerations in the x-, y- and z-axes (transversal axis (x), longitudinal axis (y), sagittal axis (z)). These mobile long-term, offline sensors are usually placed on the lateral thigh and are a well-established method of recording and assessing mobility in humans. Here accelerometers were placed on the upper trunk (midclavicular line below the right clavicle) and on the thigh (standard position when assessing mobile patients; 5 cm above the lateral knee joint gap) of immobile patients during early neurorehabilitation in order to distinguish between the following four positions: supine, side-lying right, side-lying left, sitting (Figure 2). Sensor-detected position changes were compared to standard written care documentation over 24 h. Standard care documentation is the written documentation of the passive position changes provided by the staff (nursing and therapeutic) as part of the care concept during early neurorehabilitation.

We did a qualitative and quantitative analysis of the four different positions over 24 h analyzing the duration per position, the number of position changes, and their distribution during both day and night time. Digital signal processing and data analysis were conducted using MATLAB R2012b© (MathWorks, Natick, MA, USA).

All raw data of accelerations and all visualized data sets were manually assessed and compared between sensors and written documentation for each patient over 24 h. Visualized data sets were analyzed using signals of the longitudinal (*red*) and transversal axes (*green*) for the trunk sensor and the sagittal (*blue*) and longitudinal (*red*) axes for the thigh sensor (Figure 2). To distinguish between the aforementioned four positions, two axes are necessary per sensor, namely the transversal and longitudinal axes for the trunk sensor and the sagittal and longitudinal axes for the thigh sensor. Therefore, the transversal and sagittal axis are interchangeable between the trunk and the thigh sensor, as both sensors are placed perpendicular to each other with the trunk sensor in the frontal and the thigh sensor in the sagittal plane. In detail, the tilt angles (TA) which define and distinguish the positions are given in brackets: for supine (transversal or sagittal axis/longitudinal axis: ≥0°/≥0°), for side-lying right (transversal or sagittal axis/longitudinal axis: ≥−45°/≥0°), for side-lying left (transversal or sagittal axis/longitudinal axis: ≥+45°/≥0°), or sitting (transversal or sagittal axis/longitudinal axis: ≥0°/≥−45°). Thereby, the 45-degree angle reflects the standard therapeutic positioning of neurological disabled patients in a semi-side lying position, namely a position of 45 degrees to the supine position.

**Figure 3:**
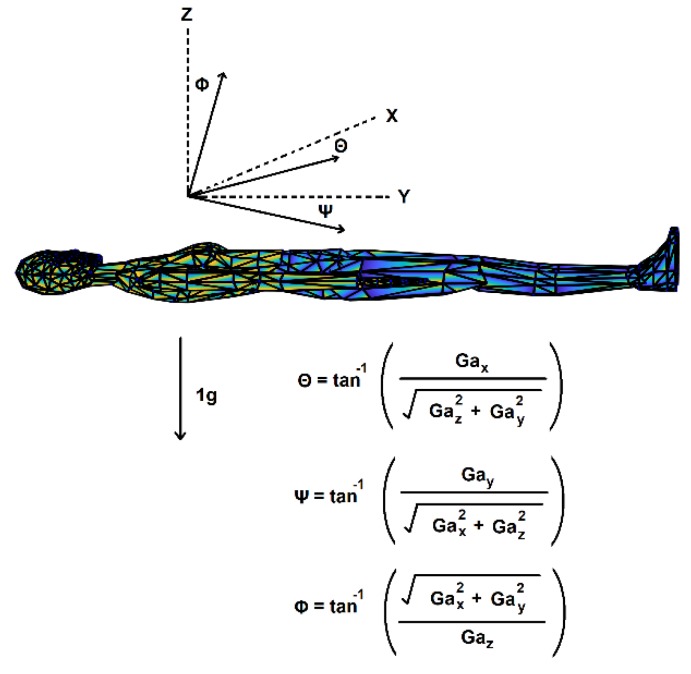
**Using an accelerometer for inclination sensing**. Angles for independent inclination sensing were computed from the gravitational component (GA) as described by Fisher [22]. Baseline tilt corrections were made based on tilt angles obtained during a supine rest position. Body segments modified from an original VRML file by Cindy Ballreich (cindy@ballreich.net) copyright 1997.

The sensors are 8-bit devices that capture linear accelerations at 20 Hz with a sensitivity of ±2 g. The sensors are initially calibrated using the proprietary activPAL^TM^ software. Tri-axial raw voltage signals were converted into gravitational units by multiplying the signal by 0.015625 g and centered to achieve zero mean. A three-point median filter was used to smoothen the resulting raw acceleration signal (RA) in each axis. A third order low pass infinite impulse response filter (IIR) with a cutoff at 0.25 Hz was applied to the raw acceleration (RA) in each axis to extract the gravitational component (GA) of the signal which corresponds to the tilt of the sensor axis with respect to the gravitation vector [21]. Tilt angles (TA) for independent inclination sensing were computed directly from the GA vectors as described by Fisher (Figure 3) [22]. The body movement/acceleration (BA) was calculated by subtracting the gravitational components from the total signal in each axis (BA = RA − GA). Baseline tilt corrections were made by subtracting the offset based on the TA obtained during supine rest position to account for sensor drift and local anatomical differences from the sensor horizontal. At supine rest position the horizontal inclination angles (θ, ψ) were set to zero and vertical inclination (φ) was set to 90 degrees.

**Figure 4:**
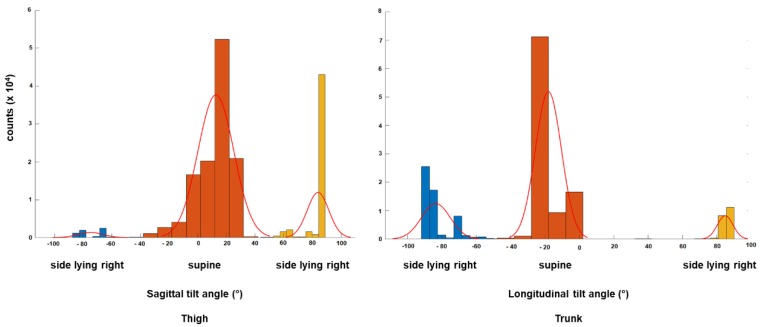
**Distribution of tilt angles in a single axis for thigh and trunk sensors during a 5 h pilot recording**. The authors’ pilot recoding shows trimodal distribution of sagittal (thigh sensor) and longitudinal (trunk sensor) tilt angles from −90° to +90° to detect position changes. An absolute angle of equal to or greater than ±45 degrees separates the three distributions and is therefore suitable to detect the three major postural transitions over the 180-degree range of side-lying right, supine and side-lying left.

**Figure 5:**
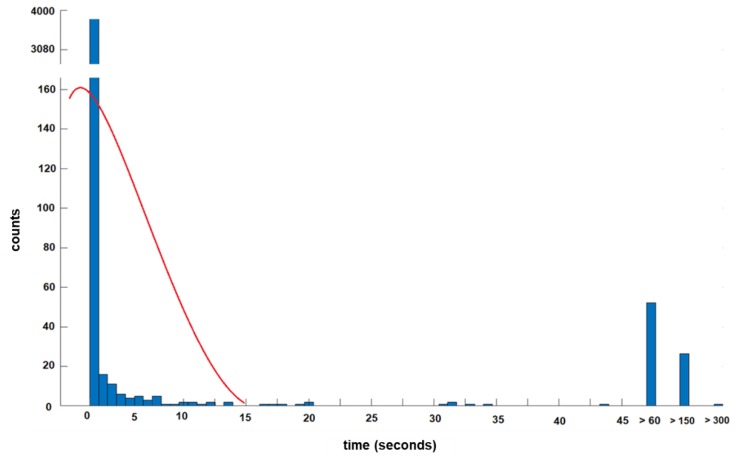
**Distribution of time intervals between Signal Magnitude Area to define the 15 s threshold**. In this 5 h pilot recording, left plots (<15 s) reflect rapid activity fluctuations and no position changes. Thus, the authors defined the cutoff at 15 s to detect position changes. Activity blocks shorter than15 s were merged with neighbor blocks using the most similar tilt angle and then, data were re-averaged to create smooth blocks. Threshold was at 0.2 g to distinguish rest and activity.

To distinguish phases of activity from resting positions, the variations of body accelerations (BA) from all three axes were combined to generate the normalized signal magnitude area (SMA). The SMA is a valid surrogate to quantify total body movements by summing up the signal energy of all three axes. The SMA incrementally increases with increasing activity, i.e., from rest to walking, exercises and extreme events such as falls. The SMA was summed up over a 1 s moving window to preserve the temporal sensitivity of the record. A fixed threshold method was used to define the activity-rest threshold at 0.2 g, which is an appropriate value and was determined after testing. Change in the horizontal TA (θ) or vertical inclination (φ) equal to or greater than 45 degrees and lasting longer than 15 s with a simultaneous activity peak at the onset of the SMA, was considered as a change in supine orientation and therefore as a transition. These artificial thresholds of 45-degree angles and the 15 s are idealized values based on the authors’ own pilot recordings over 5 h (Figure 4 and Figure 5). In detail, the defined 45-degree angle detects a transition from supine to side-lying right or left. Tilt angles were trimodally distributed over 180 degrees and an absolute angle equal to or greater than ±45 degrees detected a position change (Figure 4). Activities below 15 s reflect rapid activity fluctuations and no position change. Thus, the authors defined the 15 s cutoff, though more arbitrary, according to their pilot results (Figure 5).

Equation (1) describes the normalized signal magnitude area (SMA). In the equation BA_x_, BA_y_, and BA_z_ refer to the body component of acceleration in the x, y, and z axes, and t refers to a one second moving window (t = 1 s).

(1) $$\mathrm{SMA}=\frac{1}{t}(\int_{0}^{t}{|\mathrm{BA}}_{x}(t)|\mathrm{dt}+\int_{0}^{t}{|\mathrm{BA}}_{y}(t)|\mathrm{dt}+\int_{0}^{t}{|\mathrm{BA}}_{z}(t)|\mathrm{dt})$$

The SMA was used to divide the tilt data into blocks of activity. These data blocks are monotonous, i.e., there was no postural change between the start and the end of each block. The tilt angles within these blocks were averaged, producing an envelope tilt and fluctuations were removed. Activity or resting blocks that were shorter than 15 s were merged with the adjacent blocks with the most similar tilt angle and were re-averaged. Differences in tilt angles equal to or greater than 45 degrees were then identified as position changes while blocks without these two criteria, namely tilt angles smaller than 45 degrees and a time frame under 15 s, were ignored. Posture blocks were manually identified and defined according to the logic displayed in Figure 2.

### 2.2. Patients

Thirty out of eighty-five immobile adult patients in early neurorehabilitation were enrolled in the study. Inclusion criteria were a maximum Barthel Index of 30 and a written consent for participation in the study. Exclusion criteria were a plaster allergy and/or a dermatological illness. All subjects (10%) or their legal caregivers (90%) gave their written informed consent for inclusion before they participated in the study. The study was conducted in accordance with the Declaration of Helsinki, and the protocol was approved by the Ethics Committee of Ludwig-Maximilians University of Munich, Germany (No. 485-15).

### 2.3. Statistical Analysis

Data are presented as percentage or mean ± SEM. Additionally, trunk sensor data are presented as a cumulative duration of all 30 patients in hours and percentage per position, and as average duration per position and patient. Statistical analysis was performed using GraphPad software Prism 7.03 (GraphPad Software Inc., La Jolla, CA, USA). Groups were tested for normality using D’Agostino-Pearson test (“omnibus K2”). Group differences were analyzed using either Kruskal-Wallis-test for the non-parametric data (“data recording”) or one-way ANOVA for parametric data (“number of position changes”). Differences were considered significant, if *p* < 0.05.

## 3. Results

**Figure 6:**
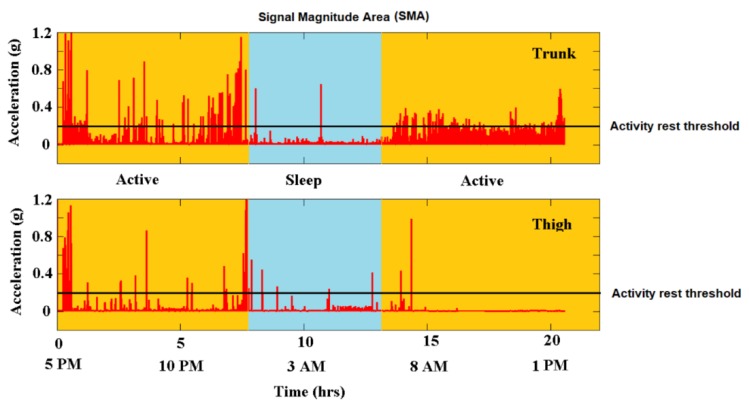
**Quantitative accelerometric data acquisition from trunk and thigh sensor**. A 53-year-old female patient suffering from left middle cerebral artery infarction and consequential right-sided hemiparesis 1.5 months prior to accelerometric measurement from upper trunk (**top**) and standard position thigh (**bottom**). The patient underwent early neurorehabilitation with a Barthel Index of 20, was wheelchair bound and needed support for position changes. This exemplary quantitative accelerometric analysis shows the advantage of upper trunk accelerometric measurement. The activity rest threshold was set at 0.2 g.

**Figure 7:**
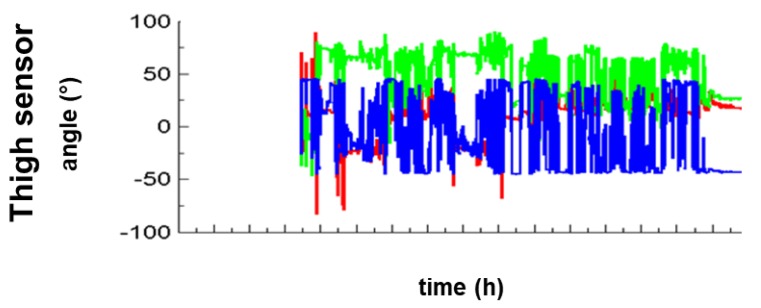
**Artifacts of thigh sensors**. Visualized accelerations in sagittal axis (*blue*) show artefacts most probably due to spontaneous leg movements, restless legs or spasticity in the cohort of immobile patients suffering from severe neurological diseases such as stroke or traumatic brain injury.

**Figure 8:**
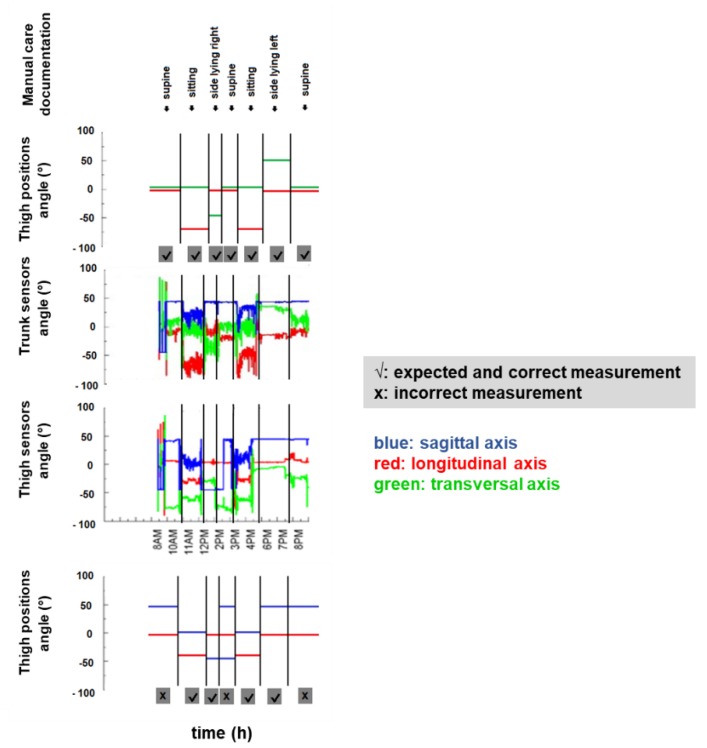
**Accelerometric data acquisition from trunk and thigh sensor**. Trunk sensors detected all position changes according to the manual care documentation and accurately distinguished supine from side-lying right/left or from a sitting position, while the thigh sensors did not detect supine position as indicated.

Accelerometric sensors stayed in place for the intended 24 h period and delivered data for the entire period. Quantitative accelerometric analysis of the thigh compared to the trunk sensors recorded fewer accelerations and were more prone to artefacts (Figure 6 and Figure 7). Trunk recording delivered data over almost the entire intended period of time, namely in 99.5 ± 0.4% of the time and was therefore better due to the completeness of data recording compared to the thigh sensor (92 ± 4.7%) (*p* < 0.0001). Trunk sensors detected 100% of the position changes documented in standard written care documentation and a few additional spontaneous position changes. Thigh sensors detected only 66% of the position changes documented in the care documentation (*p* = 0.0004) as exemplary indicated in Figure 6 and Figure 8. Based on the trunk sensor data of all 30 patients, 90% of patients (n = 27) spent more than 4 h in one position and duration per position ranged from 0 to 19.75 h. In detail, all 30 patients spent 284.75 h in supine position, i.e., 39.54% and an average of 9.49 h per patient, 110.58 h in sitting, i.e., 15.3% and an average of 3.89 h per patient, 183 h in side-lying to the left side, i.e., 25.4% and an average of 6.1 h per patient, and 138 h in side-lying to the right side, i.e., 19.2% and an average of 4.6 h per patient. On average, patients experienced six position changes per 24 h and these position changes were evenly distributed during the day and night time.

## 4. Discussion

This study provides evidence for the feasibility of using accelerometric trunk sensors to assess body position and position changes of immobile patients during early neurorehabilitation. Accelerometric trunk sensors reliably record data and are suitable for detecting position and position changes in immobile patients in comparison to the conventional sensor position on the patient’s thigh. The latter position on the patient’s lateral thigh is less suitable to assess position changes in severely and neurologically affected patients suffering from stroke, traumatic brain injuries or critical illness polyneuropathies as spontaneous and partially non-controlled leg movements are common in these patients, which might then cause incomplete data acquisition due to artifacts (Figure 7 and Figure 8). These current results suggest the value of using accelerometric trunk sensors to monitor frequency of position changes and duration of positions during and after early neurorehabilitation in order to improve patient care.

Physical activity has a significant impact on the functional outcome in neurorehabilitation. It has an impact on cognitive function, aphasia, metabolic and general health as well as in reducing mortality [1,2,3,4,5]. The patient’s mobility and positioning after discharge from the hospital may be a suitable biomarker for predicting re-hospitalization rates, which are associated with decreased functional status, depressive symptoms and reduced need for social support. Thus, mobility and position changes should become a major area of interest in overall patient health. How physical activity after discharge contributes to the patient’s well-being and prevents readmission to hospital is still insufficiently investigated [2,6,7,8,9,23]. Several reviews have emphasized that accelerometers are suitable to assess physical activity, however, mainly distinguish between walking and sedentary behavior to date. Currently, there is still a lack of standardized methods for quantifying accelerometer-assessed activity [10,12,24].

The results presented show that accelerometric trunk sensors are suitable for gaining further knowledge about the link between positioning and functional outcome in immobile patients. Accelerometric trunk sensors can reliably quantify body position changes and mobility and are desirable to continuously monitor and adjust treatment regimens during and after early neurorehabilitation. Thus, accelerometric trunk sensors have additional value as a new tool for point-of-care and off-hospital use in this immobile and care-dependent patient cohort. The use of accelerometric trunk sensors can help to show the effects of early neurorehabilitation treatment regimen after discharge and help to quantify complications and re-hospitalization rates. Thus, the implementation of accelerometric monitoring could help to evenly distribute positioning for each individual patient and to monitor the effects of positioning on outcome and complications and might be of added value in the new field of personalized medicine.

### Study Limitations

Currently automatic data reading is not available and an automatic algorithm for data acquisition needs to be established for this new method of assessing mobilization characteristics in immobile patients. In this study we used a small sample size of 30 participants and a short recording duration of 24 h to learn more about the data quality and reliability. For future assessment and investigation during hospital stays and post discharge, longer recording periods (7 or 14 days) may be more suitable [14].

In summary, our findings show the added value for the use of accelerometric sensors in severely affected neurological patients to detect body position change rates outside of a hospital. They could be used to help to adjust treatment regimens and to improve patient care. This could prevent complications in severely affected patients after discharge from hospital. Follow-up studies are needed to gain insights into the mobilization frequencies in and out of hospital. High mobilization rates may prevent complications and reduce re-hospitalization rates and this new approach could support better outcomes in the future of personalized medicine.
